# Molecular Mechanisms Linking Omega-3 Fatty Acids and the Gut–Brain Axis

**DOI:** 10.3390/molecules30010071

**Published:** 2024-12-28

**Authors:** Anna Zinkow, Wojciech Grodzicki, Malwina Czerwińska, Katarzyna Dziendzikowska

**Affiliations:** Department of Dietetics, Institute of Human Nutrition Sciences, Warsaw University of Life Sciences—SGGW, Nowoursynowska 159C, 02-776 Warsaw, Poland; ania.zinkow@gmail.com (A.Z.); wojciech_grodzicki@sggw.edu.pl (W.G.); malwina_czerwinska@sggw.edu.pl (M.C.)

**Keywords:** cognitive function, docosahexaenoic acid (DHA), eicosapentaenoic acid (EPA), gut microbiota, gut–brain axis (GBA), hypothalamic–pituitary–adrenal (HPA) axis, inflammation, omega-3 (n-3) fatty acids

## Abstract

The gut–brain axis (GBA) is a complex communication network connecting the gastrointestinal tract (GIT) and the central nervous system (CNS) through neuronal, endocrine, metabolic, and immune pathways. Omega-3 (n-3) fatty acids, particularly eicosapentaenoic acid (EPA) and docosahexaenoic acid (DHA), are crucial food components that may modulate the function of this axis through molecular mechanisms. Derived mainly from marine sources, these long-chain polyunsaturated fatty acids are integral to cell membrane structure, enhancing fluidity and influencing neurotransmitter function and signal transduction. Additionally, n-3 fatty acids modulate inflammation by altering eicosanoid production, reducing proinflammatory cytokines, and promoting anti-inflammatory mediators. These actions help preserve the integrity of cellular barriers like the intestinal and blood–brain barriers. In the CNS, EPA and DHA support neurogenesis, synaptic plasticity, and neurotransmission, improving cognitive functions. They also regulate the hypothalamic–pituitary–adrenal (HPA) axis by reducing excessive cortisol production, associated with stress responses and mental health disorders. Furthermore, n-3 fatty acids influence the composition and function of the gut microbiota, promoting beneficial bacterial populations abundance that contribute to gut health and improve systemic immunity. Their multifaceted roles within the GBA underscore their significance in maintaining homeostasis and supporting mental well-being.

## 1. Introduction

The gastrointestinal tract (GIT) plays a key physiological role in nutrient digestion and absorption but is also particularly vulnerable, since the alimentary canal is constantly exposed to external and potentially hazardous factors [1]. Given this dual character, GIT has to be closely monitored by the organism’s master controller—the brain [2]. The exchange of information between the intestines and the central nervous system (CNS) is mutual. It involves a plethora of neuronal, endocrine, metabolic, and immune factors, known collectively as the gut–brain axis (GBA). The GBA is crucial for maintaining homeostasis and mental health [3]. The pathways that form the axis include interactions with the nervous system as well as molecular signals, like microbial metabolites, tight junction protein expression, and cytokines released during inflammation [4]. Dietary factors, particularly omega-3 polyunsaturated fatty acids (PUFAs), significantly modulate the GBA by influencing gut microbiota composition, enhancing intestinal barrier integrity, and supporting neural function, showing the interplay between nutrition and gut–brain health. Based on the position of the first double bond relative to the terminal end of the carbon chain, PUFAs can be classified into two main groups: omega-3 (n-3) and omega-6 (n-6) fatty acids [5]. n-3 Fatty acids are crucial dietary fats derived from plant sources and marine organisms. α-linolenic acid (ALA, C18:3), an 18-carbon chain fatty acid, is the shortest in the n-3 family and is classified as a short-chain polyunsaturated fatty acid (SC-PUFA). It is also the most abundant n-3 fatty acid found in dietary sources, such as flaxseed, chia seeds, walnuts, and rapeseed oil [6]. ALA plays unique roles supporting cardiovascular health by reducing lipids, blood pressure, and inflammation. Additionally, ALA-derived oxylipins promote vascular health, and emerging evidence highlights its potential in improving cognitive function and supporting brain health [7]. Marine n-3 fatty acids, including eicosapentaenoic acid (EPA, C20:5) and docosahexaenoic acid (DHA, C22:6), are classified as a long-chain PUFAs (LC-PUFA) and exhibit greater biological activity than their plant-derived counterparts [8]. These essential fatty acids cannot be synthesized in the human body and must be supplied through the diet [9]. LCPUFAs, including EPA, DHA, and arachidonic acid (AA, C20:4, n-6 fatty acid), are major components of cellular membrane phospholipids (75–88%, depending on the cell type). Reduced dietary intake of n-3 fatty acids, such as ALA, EPA, and DHA, can lead to decreased content of those fatty acids in brain cells and organelles. DHA, in particular, is the most abundant n-3 fatty acid in the CNS, and is especially concentrated in the membrane lipids of gray matter [8,10]. Therefore, it is to be expected that n-3—and LCPUFAs in particular—would play a crucial role in the GBA, maintaining proper functioning of both the gut and the brain.

## 2. The Gut–Brain Axis: Definition and Overview

The fundamental part of the GBA is the enteric nervous system (ENS), which entails 100 million nerve cells located in two principal plexuses: the submucosal and the myenteric plexuses [11,12]. ENS forms part of the autonomic nervous system (ANS) and regulates processes related to digestion, nutrient absorption, the release of gastrointestinal hormones, and peristalsis. It serves as an intermediary between the GIT and the CNS by detecting diverse stimuli originating from the intestinal lumen and transferring them to the brain [11,12,13]. The bidirectional communication network between the CNS and the GIT involves the ENS and parasympathetic innervation, primarily through the vagus nerve fibers that innervate most of the GT. The vagus nerve, also known as the tenth cranial nerve, plays a central role in the complex communication network between the gut and the brain. This nerve is a branch of the ANS that connects the CNS to the ENS through both afferent and efferent nerve fibers [11,12,13]. Given their multifarious receptors, the constituents of the vagus nerve are sensitive to diverse stimuli, including mechanical tension, hormones, and other chemical incentives. The signals they provide are integrated by the solitary nucleus in the brainstem and elicit a wide array of effects in the brain, stimulating regions related to feeding behavior, anxiety, or emotions [11]. On the other hand, efferent vagal activity has an impact on the gut environment, influencing the immune system and metabolism [11,12].

The CNS also communicates with the GIT through the hypothalamic–pituitary–adrenal (HPA) axis, a key component of the gut–brain communication pathways [11]. The HPA axis activity is initiated by the release of corticotropin-releasing hormone (CRH) from the hypothalamus, which stimulates the anterior pituitary to secrete adrenocorticotropic hormone (ACTH). ACTH, in turn, stimulates the adrenal cortex to release cortisol, a steroid hormone, which plays an important role in regulating metabolism, immune response, and maintaining homeostasis under stress conditions [4,14]. The endocrine factors that influence and regulate the functioning of the GBA include CRH and cortisol. The latter can affect the functioning of immune cells, leading to the production of both proinflammatory cytokines (e.g., tumor necrosis factor-α (TNF-α), interferon-γ (INF-γ), and interleukin 6 (IL-6)) and anti-inflammatory cytokines (e.g., IL-10). Additionally, cortisol increases the permeability of the intestinal barrier, allowing bacterial antigens to pass from the intestinal lumen into the bloodstream, which contributes to the development of systemic inflammation [15].

Gut-associated lymphoid tissue (GALT), which consists of immune cells, is another component of the GBA, positioned at the interface between gut contents and the internal environment of the body [16]. It constitutes 70–80% of the human immune system, highlighting the crucial and sensitive role the intestines play in the body’s defense mechanisms [17]. M cells and dendritic cells interact with luminal antigens, activating T and B lymphocytes in Peyer’s patches [1]. Cytokine release from immune cells and enterocytes triggers an immune response that can extend beyond the GI tract, reaching the CNS via the bloodstream and affecting vagus nerve signaling. The immune cells’ defense is complemented by the protective function of the intestinal barrier [1,11]. The intestinal mucosa functions as a physical and immunological defense barrier, consisting of key components, such as the outer mucus layer with commensal microbiota, antimicrobial peptides, and secretory immunoglobulin A. It also includes a central single layer of epithelial cells and an inner lamina propria containing innate and adaptive immune cells like T cells, B cells, macrophages, and dendritic cells [18].

GALT is also a very important site of interaction with microbial agents and their metabolites [11,16]. Collectively known as the gut microbiota, these microorganisms significantly influence the host’s physiology [19]. Interestingly, the number of microbes within the digestive tract exceeds the number of human cells by a factor of 1.3, which highlights their essential role in both health and disease [20,21]. Intestinal microorganisms produce a number of neurotransmitters and neuromodulators—including serotonin, melatonin, γ-aminobutyric acid (GABA), catecholamines, and histamine—which contribute significantly to the proper functioning of brain areas involved in emotion processing, motor activity, and cognitive skills [21,22]. Intestinal microorganisms synthesize and metabolize tryptophan, producing about 95% of systemic serotonin in the gut, which also participates in gut–brain communication [23,24]. Current research demonstrates that the microbiota not only interacts with the CNS but also shapes its development by influencing growth and maturation of brain cells [11,16,25]. Additionally, alterations in microbiota composition have been associated with the onset of various CNS-related disorders, such as Alzheimer’s disease, Parkinson’s disease, autism, and depression [12].

GALT and gut microbiota collaboratively reinforce the intestinal barrier, contributing to immune protection and maintaining gut homeostasis. Epithelial cells are the primary physical components of the intestinal barrier [26]. Due to the impermeability of cell membranes to hydrophilic solutes without specific transporters, the passage of such molecules through intestinal epithelial cells (IECs) is highly restricted. Lipophilic or larger molecules are primarily absorbed via diffusion and endocytosis that is controlled by junctional complexes, with the most crucial being tight junctions (TJs), adherens junctions (AJs), and desmosomes. TJs, located at the apical region, seal the intercellular space and include proteins such as claudins, occludin, and zonula occludens (ZO)-1 and ZO-2. AJs are positioned below TJs and, together with desmosomes, maintain the epithelial integrity through strong adhesive bonds [27]. The proper functioning of the intestinal barrier is ensured by the tight adhesion of these cells, allowing for transcellular transport and enabling selective absorption.

The GBA also includes the physical barrier that protects the CNS. The blood–brain barrier (BBB) is formed by endothelial cells that are connected by protein junctions [28]. These specialized squamous epithelial cells create a single layer of polarized lining on the inner side of the capillary wall [29]. The intercellular spaces of the capillary endothelium are covered by a dense network of high-resistance junctions, with TJs being the most significant ones. The main components of these TJs are the proteins occludin and claudin [30]. Another set of proteins found in the barrier junctions of the brain are cell adhesion molecules— junctional adhesion molecules (JAM)-A, -B, -C, and -D—with JAM-A believed to influence TJs formation. Along with membrane proteins, the barrier connections also include cytosolic proteins such as ZO-1, -2, and -3 [31]. Additionally, astrocytes contribute to the neurovascular cell complex by forming an extra barrier that circulating blood compounds must cross to reach the brain, enhancing the selectivity of the BBB during periods of increased neuronal activity. Transport of substrates and metabolites is carefully regulated by membrane systems, including the sodium–potassium pump. The BBB serves as a protective barrier against neuroactive substances (e.g., catecholamines) and blood-borne toxins, while also supplying neurons with essential nutrients like glucose and amino acids. Moreover, this barrier protects also against the entry of immune system cells [30].

**Figure 1:**
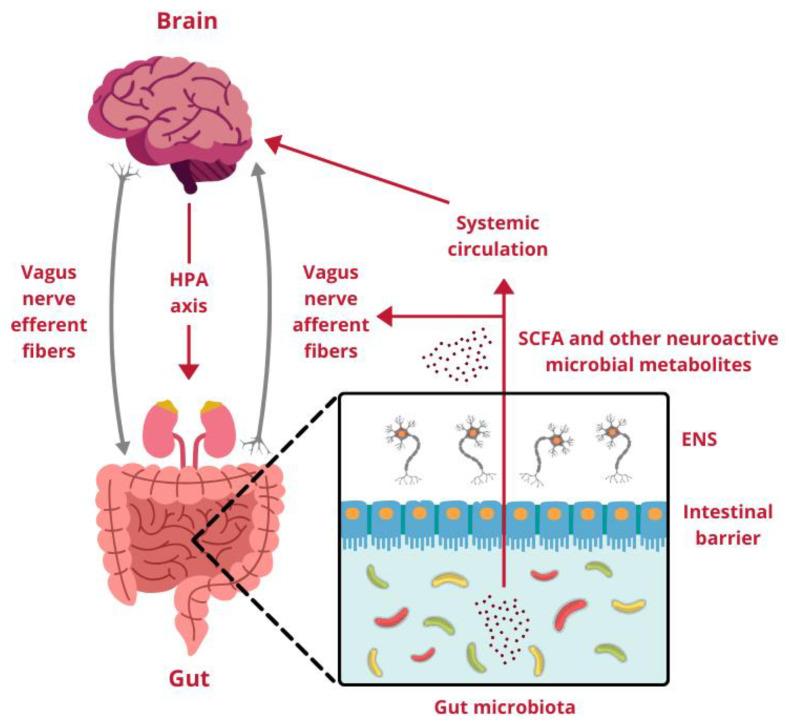
Interaction Between the gut and the brain through the gut–brain axis. HPA axis, hypothalamic-pituitary-adrenal axis; ENS, enteric nervous system; SCFA, short-chain fatty acids. Designed using elements by ©Canva, sparklestroke, Pixeden, iconsy, OpenClipart-Vectors via Canva.com (access date: 18 November 2024).

In summary, multiple communication pathways link the gut and the brain, encompassing systems like the ANS, HPA axis, ENS, intestinal barrier, GALT, microbiota, and the BBB. These systems engage in continuous interaction and information exchange within the GBA (Figure 1) [13].

## 3. Molecular Mechanisms Linking n-3 Fatty Acids, Microbiota, and Brain Function

### 3.1. Omega-3 Fatty Acids as Components of Cell Membranes

**Figure 2:**
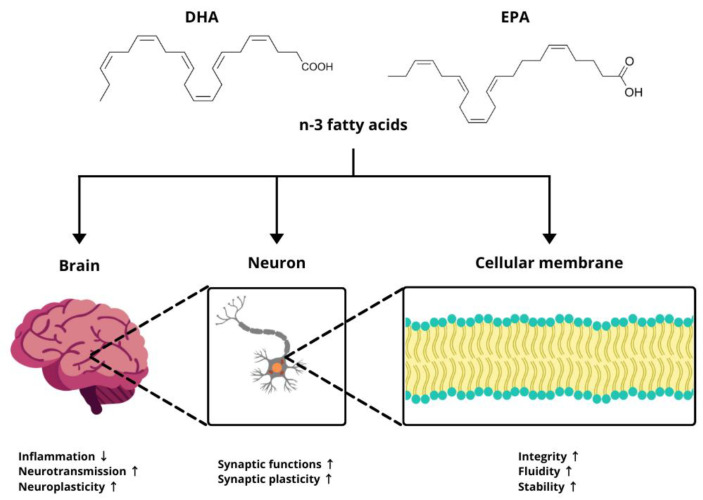
The role of omega-3 fatty acids as components of cell membranes. ↑ indicates an increase, ↓ indicates a decrease. Designed using elements by ©Canva, sparklestroke, Pixeden, iconsy, OpenClipart-Vectors via Canva.com (access date: 18 November 2024).

The multifaceted roles of n-3 fatty acids within biological systems include complex interactions with the GBA. They are integral components of phospholipids in the nerve cell membranes, which enhance their structure and are crucial for their optimal fluidity [32]. This fluidity influences neuronal information transfer by affecting neurotransmitter binding and the speed and integrity of cell signaling [33,34,35]. DHA represents approximately 15% of all fatty acids in the gray matter of the prefrontal cortex, while EPA and n-3 docosapentaenoic acid (DPA) account for only ~1% of brain fatty acids [36,37,38]. However, the hydroxy derivative of DHA (2-hydroxydocosahexaenoic acid, OHDHA) has demonstrated therapeutic potential in treating Alzheimer’s disease in a mouse model. Administration of this DHA derivative increased brain DHA levels and reduced Aβ (amyloid-β) levels as well as Aβ-induced tau phosphorylation—key factors in the progression of the disease. These effects supported neuronal cell membranes, preserving proper synaptic function, which is essential for signaling and membrane stability in the CNS [39]. The mechanism through which neuronal fluidity changes is based on the displacement of cholesterol from the membrane as well as the induction of non-lamellar structure formation in the membrane [40,41,42]. Different lipid concentrations in the cell membrane can alter its fluidity as well as the structure and functioning of embedded proteins, such as enzymes, receptors, and ion channels. It is believed that the incorporation of fatty acids into cell membranes also affects the inflammatory cellular responses [43,44,45]. EPA and DHA compete with dihomogammalinolenic acid and AA for incorporation into the phospholipid membrane, sharing enzymes involved in the eicosanoid production process [46,47]. Metabolic transformations of DHA and EPA carried out by cyclooxygenases (COX), lipoxygenases (LOX), and cytochrome P450 enzymes result in the production of numerous eicosanoids and docosanoids [48,49]. These metabolites influence the brain-derived neurotrophic factor (BDNF), increasing synaptic plasticity and enhancing neurotransmission, thus providing neuroprotective effects, as demonstrated in studies conducted on the multipotent human hippocampal progenitor cell line HPC0A07/03C and the human bone marrow neuroblastoma SH-SY5Y cell line (multipotent human hippocampal progenitor cell line) [48,49,50]. Both animal and human studies indicate that the incorporation of EPA and DHA into cell phospholipids during inflammatory processes is dose-dependent and occurs at the expense of AA content [47,51,52,53,54,55,56]. The role of n-3 fatty acids as components of CNS cellular membranes is summarized in Figure 2.

### 3.2. Impact of Omega-3 Fatty Acids on Inflammation

The modulation of inflammation by n-3 fatty acids, mainly through their effects on eicosanoid production, illustrates a critical mechanism by which dietary fats influence both neurological health and systemic inflammatory responses [57,58]. Prostaglandins, thromboxanes, leukotrienes, and hydroxyl and hydroxy fatty acids are enzymatic metabolic products of PUFA, known as eicosanoids. These compounds play a pivotal role in the inflammatory processes and neurological pathways within the GBA [59,60]. The structural differences in eicosanoids derived from EPA and AA affect the biological activity of EPA derivatives, as eicosanoid receptors have a lower affinity for the mediators derived from EPA than ARA [61]. An in vitro study on HEK293 cell lines demonstrated 50–80% lower activity of prostaglandin E3 (PGE3) compared to prostaglandin E2 (PGE2) on prostaglandin E2 receptors 1, 2, 3, and 4 (EP1, EP2, EP3, and EP4), indicating nuanced influence of dietary fats on neuroinflammatory responses [62]. Eicosanoids derived from EPA and DHA have anti-inflammatory properties [47,55,58,63]. EPA competes with AA for the cyclooxygenase enzyme system, effectively inhibiting the production of proinflammatory eicosanoids from AA. Both DHA and EPA reduce the release of proinflammatory cytokines, such as interleukin-1β (IL-1β), -2 (IL-2), and -6 (IL-6), along with IFN-γ and TNF-α [57,63,64,65]. These cytokines can affect the CNS both indirectly and directly by reducing the availability of neurotransmitter precursors, influencing their metabolism, transport, and regulation, as well as impacting the HPA axis and mRNA-encoding proteins involved in neurotransmitter metabolism [66,67,68,69]. Flaxseed oil inhibits the synthesis of proinflammatory cytokines, thereby regulating neurotransmitter production and the HPA axis [70]. Marine-derived n-3 fatty acids lead to the production of pro-resolving lipid mediators, such as EPA-derived resolvins (E series), DHA-derived resolvins (D series), and resolvins derived from DPA n-3 (RvDn-3 DPA), along with protectins (also known as neuroprotectins when produced in the nervous tissue) and maresins derived from DHA. Their synthesis occurs through COX and LOX pathways, acting intercellularly [57,71,72,73,74]. The anti-inflammatory actions of n-3 fatty acids involve binding to peroxisome proliferator-activated receptors (PPARs), G-protein–coupled receptor 40 (GPR40), and free fatty acid receptor 4 (FFA4), also known as GPR120, which promote the production of anti-inflammatory lipids—resolvins and protectins. These lipids play a crucial role in inhibiting the activation of key inflammatory regulators, including transcription factor nuclear factor kappa B (NF-kB), IL-1 β, and TNF-α release [75,76,77,78,79]. Supplementation with EPA and DHA in mice has been shown to increase the number of T lymphocytes [80]. Additionally, DHA and EPA are able to inhibit IL-6 and interleukin 8 (IL-8) production stimulated by lipopolysaccharide (LPS) in human endothelial cells. Moreover, EPA has been observed to inhibit TNF-α production by cultured monocytes [81,82]. LC-PUFAs also reduce LPS-induced proinflammatory cytokine production in human blood monocytes and in murine fetal liver-derived macrophages, leading to a decrease in TNF-α level in serum, NF-kB activation, and IL-1 β production by monocytes [82,83,84]. This reduction can trigger the release of substantial amounts of anti-inflammatory factors, such as interleukin-10 (IL-10), from resident macrophages. LC-PUFAs play a critical role in directly modulating cytokine production and immune cell regulation. They also exert broader anti-inflammatory effects through key intracellular signaling pathways, such as NFκB, that influence overall immune response and inflammatory status mainly due to the increased level of inflammatory cytokines, adhesion molecules, and cyclooxygenase-2 (COX-2) [82,85,86]. NFκB is activated by the signaling cascade triggered by external inflammatory stimuli, including endotoxin binding to toll-like receptor (TLR) 4. Upon activation, the NFκB dimer is translocated to the nucleus, where it upregulates gene expression [87,88,89,90].

**Figure 3:**
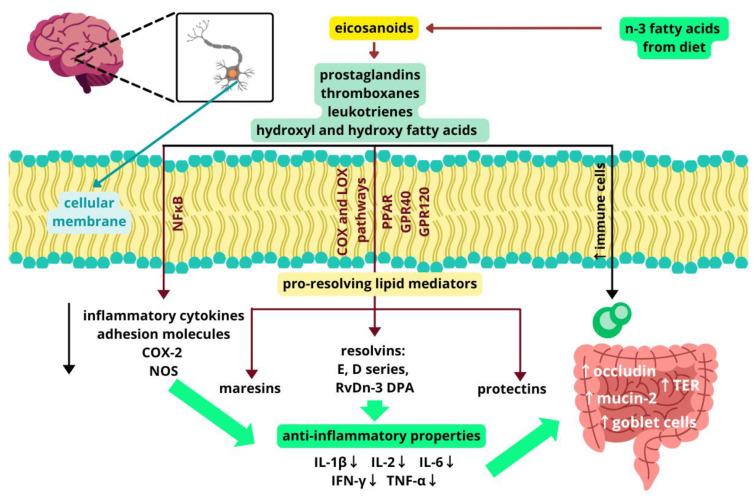
Anti-inflammatory role of n-3 fatty acids. NF-kB—nuclear factor kappa B; COX-2—cyclooxygenase-2, prostaglandin-endoperoxide synthase 2; LOX—lipoxygenase; NOS—nitric oxide synthase; PPAR—peroxisome proliferator-activated receptors; GPR40—G-protein–coupled receptor 40; GPR120—G-protein–coupled receptor 120; RvDn DPA—resolvins derived from DPA n-3; Il-1β—interleukin 1β; IL-2—interleukin 2; IL-6—interleukin 6; INF-γ—interferon-γ; TNF-α—tumor necrosis factor α; TER—trans-epithelial electrical resistance. ↑ indicates an increase, ↓ indicates a decrease. Designed using elements by ©Canva, sparklestroke, Pixeden, iconsy, OpenClipart-Vectors via Canva.com (access date: 18 November 2024).

The documented anti-inflammatory and regulatory capabilities of EPA and DHA on cytokine dynamics and cellular mediator pathways also help preserve the integrity of cellular barriers, such as the BBB, by reducing inflammation-induced disruption and enhancing barrier functions. Transmembrane proteins, including claudin-5 and occludin, along with scaffold proteins, like ZO-1 and ZO-2, participate in the tight junction complex of endothelial cells in the CNS [91,92]. LC-PUFAs indirectly enhance the integrity of the BBB and reduce its permeability by modulating levels of inflammatory cytokines [93,94]. Systemic administration of IL-1β has been demonstrated to cause prolonged BBB disruption, activate matrix metalloproteinase-9, and induce rearrangement of claudin-5 in cerebral vessels following transient middle cerebral artery occlusion in mice [95]. Therefore, it is suggested that n-3 fatty acids may strengthen the integrity of the BBB by downregulating the expression of proinflammatory cytokines. Interestingly, research indicates that certain anti-inflammatory cytokines, including IL-4, IL-10, and IL-13, can also contribute to damaging the BBB, but the precise mechanisms behind these effects are not yet well understood [96]. However, the disruption kinetics of the barrier via IL-4 seems to be similar to that of n-6 PUFAs, such as di-homo-gamma-linolenic acid (DGLA, C20:3 n-6) and AA. In comparison n-3 PUFAs, including EPA and DHA, support epithelial barrier integrity by enhancing trans-epithelial electrical resistance (TER) and significantly reducing IL-4-induced permeability. This suggests that LC-PUFAs play a crucial role in maintaining barrier function, as demonstrated by Willemsen Le et al. [97]. Tight junction complexes actively control paracellular permeability and are sensitive to soluble barrier-disrupting mediators [98,99]. Proteins such as occludin, ZO-1, and claudins play critical roles in regulating the intestinal barrier, controlled by the perijunctional actomyosin ring and myosin light chain kinase [100,101,102]. LC-PUFAs improve trans-epithelial resistance, but the exact mechanism of its impact on this process is not fully understood. Several mechanisms have been proposed to explain the effects of n-3 LC-PUFAs on BBB integrity, particularly their roles in modulating inflammatory pathways and enhancing antioxidant defense systems. Notably, n-3 PUFAs suppress IFN-γ-induced expression of TNF-α, IL-6, nitric oxide synthase (NOS), and COX-2, while promoting the upregulation of heme oxygenase-1 (HO-1) in BV-2 microglia cells. BBB disruption and “leaky gut” phenomenon increase the transfer of neurotoxins into the brain, leading to elevated production of proinflammatory molecules, reactive oxygen species, and increased bacterial adhesion through receptors, thereby disrupting endothelial connections [103]. n-3 Fatty acids, specifically EPA and DHA, have been shown to enhance basal trans-epithelial resistance (TER), thereby reducing the permeability of the gut barrier in human intestinal epithelial cells (T84) in vitro, indirectly through mechanisms involving IL-4. LCPUFAs, such as dihomo-γ-linolenic acid (DGLA), AA, EPA, and DHA, stimulate basal resistance and mitigate IL-4-induced permeability changes [97,104,105]. Supplementation with EPA in rats has been shown to increase the expression of occludin, a protein playing an important role in the intestinal barrier integrity [106,107,108]. These protective effects are associated with support and protection of the tight junction structure and function. Additionally, n-3 fatty acids have been shown to increase the number of goblet cells, promote the growth of beneficial bacteria, and elevate the expression of mucin 2, all of which contribute to improved intestinal barrier integrity [104,109]. Summarized anti-inflammatory effects of n-3 fatty acids in the gut and the brain are shown in Figure 3.

### 3.3. Impact of Omega-3 Fatty Acids on the Nervous System and Cognitive Functions

Omega-3 fatty acids, mainly DHA and EPA, are essential for maintaining neural integrity and improving cognitive functions, as they serve as integral components of neuron membrane structure and play a critical role in neurogenesis, neural signaling, and neuroprotection. DHA also promotes neuron growth by enhancing protein kinase B signaling [110]. This process requires the accumulation of lipids in newly formed membranes and DHA facilitates the incorporation of cholesterol into structures important for myelination and neurite extension, thereby assisting in the organization of the lipid raft domain of the membrane [111,112,113]. Summarized data presenting the effects of n-3 fatty acids on cognitive functions are presented in Table 1.

Neurogenesis is essential for cognitive function, as it supports brain plasticity and adaptability, fundamental for learning and memory retention. This process is most apparent during prenatal development, but it persists in certain parts of the adult brain, most notably in the hippocampus, a structure crucial for learning and memory formation [114,115]. BDNF is a protein that belongs to the neurotrophin family of growth factors, which are vital for the growth, survival, and differentiation of neuron cells. It plays a key role in neuroplasticity, and it is a critical mediator that links neurogenesis to broader brain functions, particularly those related to learning, memory, and overall cognitive health [116,117,118]. n-3 Fatty acids—especially DHA—increase BDNF levels, thereby influencing the growth, differentiation, and viability of neurons in the hippocampus, playing a significant role in the regulation of various neurotransmitter systems [119,120,121,122]. Direct administration of BDNF to the dorsal hippocampus in rats significantly increased the granule cell layer of the hilar region [123]. Deuterated polyunsaturated fatty acids (D-PUFAs) supplementation has been shown to alleviate cognitive decline through its antioxidant activity and reduction in lipid peroxidation in a Huntington’s disease mouse model [124]. In an animal model of spinal cord injury, DHA treatment following injury led to comprehensive functional improvements, evidenced by enhanced fore and hindlimb locomotion and better motor control in the grid exploration and staircase tests. This was accompanied by structural neural adaptations, including increased synaptophysin density around motor neurons, enhanced cortical synaptogenesis, amplified serotonergic innervation, and increased sprouting of corticospinal axons in both rostral and caudal regions of the injured area [125]. DHA also promotes hippocampal neuron development in vitro by supporting neurite growth and branching and also synaptogenesis. Moreover, in vivo DHA depletion in the fetal hippocampus resulted in inhibition of hippocampal neuron development in culture, which can be reversed with DHA supplementation [126].

n-3 Fatty acids increase synaptic plasticity in hippocampal neurons and improve its glutaminergic activity, which is crucial for cognitive function as it mediates synaptic plasticity and neurotransmission, fundamental for learning, memory formation, and overall brain cell connectivity [127,128,129]. A 6-month randomized controlled trial showed that supplementation at a dose of almost 2 g of EPA and DHA decreases depression symptoms in the Geriatric Depression Scale in geriatric patients and DHA improves cognitive function, i.e., verbal fluency tested with Initial Letter Fluency [130]. In a model of accelerated aging with prediabetic status, dietary enrichment with flaxseed and fish oils enhanced spatial learning and working memory performance in the Morris water maze test through reduction in inflammatory markers and toxic metabolites in the CNS of male rats. Elevated n-3 fatty acids levels in the frontal cortex following supplementation were associated with protective effects against cognitive impairment and reduced depressive-like behaviors linked to gray matter atrophy, suggesting a crucial role of EPA in preventing cognitive decline [131].

The cognitive enhancements provided by n-3 fatty acids primarily stem from their profound impact on cellular structures, which leads to the modulation of the physical aspects of brain health, particularly the stabilization of neuronal membranes and ion channels [132,133,134]. In a randomized, double-blind, controlled trial, healthy older adults who consumed 3.7 g per day of flaxseed oil containing 2.2 g of ALA over 12 weeks demonstrated improved verbal fluency. This improvement is believed to result from changes in neuronal cell membrane structure across broad anatomical regions, enhanced membrane fluidity, and improved intercellular connectivity, which typically decline with age [135]. LC-PUFAs influence the integrity of membrane proteins, including enzymes, receptors, and ion channels. They regulate membrane protein integrity, affecting enzymatic activity, receptor function, and ion channel conductance. Administration of DHA demonstrated neuromodulatory effects via ion channel regulation, subsequently reducing the amplitude of epileptiform discharges in experimental rodent models of seizure activity [134,136]. These changes occurred through sodium channel blockade and neuronal membrane stabilization by suppressing calcium-gated membrane tension, thereby blocking synaptic transmission [132,133,134,136,137,138]. Research indicates that n-3 LC-PUFAs play a crucial role in promoting brain health. They enhance neuroprotective capabilities, support neuroplasticity, and reduce neuroinflammation, all of which are essential for sustaining cognitive functions. Short-term exposure to EPA and DHA reversed spatial working memory deficits in older mice by reducing IL-1β expression in inflammation-associated hippocampi [139]. Similarly, a high-fat diet enriched with flaxseed oil improved spatial learning and working memory in male mice during the Morris water maze test by reducing levels of inflammatory markers and toxic metabolites in the CNS [140].

n-3 Fatty acids contribute to brain health by modulating membrane structures and reducing neuronal damage. Additionally, they directly impact cellular mechanisms that eliminate harmful proteins and facilitate cellular repair, highlighting their potential therapeutic roles in neurodegenerative diseases [141]. DHA and EPA stimulate and increase the expression of insulin-degrading enzyme (IDE) genes, which raises the levels of IDE, the main enzyme responsible for degrading amyloid-beta (Aβ) peptide secreted into the extracellular space of neuronal and microglial cells [142]. The accumulation of Aβ peptides is linked to the development of AD, and the effect of n-3 fatty acids may assist in removing these peptides from the brain and modulating inflammation, thereby supporting brain glial cells [143,144]. Additionally, DHA exhibits a protective effect on dopaminergic neurons in a Parkinson’s disease animal model induced by the neurotoxin 1-methyl-4-phenyl-1,2,3,6-tetrahydropyridine (MPTP) [142]. Moreover, another study demonstrated that DHA administration significantly increased TH-positive neurons compared to the control group when exposed to MPTP, suggesting changes in dopaminergic activity [145]. It has been suggested that the DHA may delay or slow the progression of Parkinson disease development by blocking the conversion of MPTP to MPP+ (methylpyridinium ion) or by preventing the uptake of MPP+ into dopaminergic terminals. Furthermore, DHA has been shown to influence synaptic plasticity and cognitive functions by activating syntaxin 3, a plasma membrane protein that plays a significant role in membrane expansion [146,147].

**Table 1:** Effects of n-3 fatty acids on the CNS and cognitive functions.

Models	Type of Study	Source and Dose	Exposure	Effect Related to Nervous System and Cognitive Functions	Reference
In vitro model studies					
Embryonic neurons from E18 mouse hippocampi pregnant C57/BL6 mice	in vitro	Diet with 2.5 wt% of linolenic acid plus 0.9 wt% DHA	16 days	- Increased neurite growth and synaptogenesis - Enhance glutamatergic synaptic activity	[126]
Hippocampus of Sprague Dawley rats	in vitro	50 μM of DHA	Single dose	- Attenuation of epileptic activity	[136]
In vivo model studies					
Q140 mouse model of Huntington’s disease	in vivo	Deuterium-reinforced D_2_-Lin (KI D-PUFA)	5 months	- Alleviation of cognitive decline	[124]
Female Sprague Dawley rats	in vivo	5 mL/kg of DHA (i.v. injection) post-injury	Single bolus	- Improve locomotion and BBB score - Enhanced synaptogenesis in cortical neurons	[125]
Male Sprague Dawley rats with prediabetic status	in vivo	2% fish oil (EPA + DHA) or 2% flaxseed oil	3 months	- Improved spatial memory	[131]
Adult male Sprague Dawley rats	in vivo	Diet with n-6/n-3 PUFA ratio at 6:1 (1.25% DHA, 0.25% EPA)	12 days	- Enhanced synaptic function underlying learning and memory - Improved spatial learning	[146]
C57Bl6/J mice 22 months old (aged)	in vivo	EPA and DHA from tuna oil	2 months	- Inhibition proinflammatory cytokine expression - Prevention of morphological alterations in hippocampal tissue - Amelioration of spatial memory impairments	[139]
Obese male C57BL/6 mice 8 weeks old	in vivo	n-3 PUFA from linseed oil	16 weeks	- Improved spatial memory - Reduced inflammatory markers (TNF-α) - Decreased toxic metabolite levels in the CNS	[140]
Male C57BL/6 mice model of Parkinson’s disease (3 months old)	in vivo	36 mg/kg/d of DHA (in corn oil)	30 days	- Decrease levels and activity of heme oxygenase (HO) in substantia nigra- Decrease in levels of Nuclear Factor E2-related factor 2, HO-1 and HO-2 in substantia nigra	[148]
Male C57BL/6 mice model of Parkinson’s disease (10 months old)	in vivo	36 mg/kg/d of DHA (in corn oil)	30 days	- Protection against oxidative stress - Significant increase in TH-positive neurons	[145]
Human subject studies					
Healthy older adults 65–80 years old	human subjects	3.7 g/day of flaxseed oil with 2.2 g of alpha-linolenic acid	12 weeks	- Improve lexical fluency	[135]
Elderly people (over 65 years old)	human subjects	1.67 g EPA + 0.16 g DHA/d or 1.55 g DHA + 0.40 g EPA/d	6 months	- Decrease in depression symptoms	[130]

### 3.4. Impact of Omega-3 Fatty Acids on the HPA Axis

As a neuroendocrine factor, cortisol is essential in the stress response and plays a critical role in regulating GBA functions. As a primary stress hormone released by the adrenal glands in response to stress, cortisol forms part of the body’s HPA axis, which is closely connected to the GBA. Dysregulation of this system, particularly HPA axis hyperactivity, has been implicated in the pathophysiology of anxiety, depression, and other stress-related conditions [149,150]. n-3 fatty acids, particularly EPA and DHA, have been shown to regulate the HPA axis by reducing excessive cortisol production. Research has shown a reduced cortisol response to acute mental stress in healthy men who were given 7.2 g/day of fish oil for 3 weeks [151]. Preclinical and clinical data indicate that low plasma levels of n-3 fatty acids are correlated with higher CRH [149] and plasma concentration of cortisol [152,153], while n-3 fatty acid supplementation may decrease CRH expression and corticosterone secretion [154,155]. CRH, crucial in regulating the HPA axis, is produced in neurons within the hypothalamic paraventricular nucleus (PVN), which receives input from the limbic system and the brainstem. This connectivity enables these neurons to respond to both psychological and physical stressors [156]. In vitro studies confirmed that n-3 fatty acid-deficient rats had exaggerated distress behaviours in comparison with rats with the appropriate n-3 fatty acid levels during administration of CRH and were normalized upon restoration of n-3 fatty acid levels [157]. Studies conducted on male Finnish psychiatric patients (with diagnosed depression, alcoholism, or both) and healthy patients without psychiatric disorders have also shown that excessive stress response and HPA hyperactivity may be related to concentrations of brain and plasma neuroactive steroids (NASs) [158]. NASs are produced in the CNS from cholesterol and have the ability to alter neuronal excitability rapidly. In a study carried out on male psychiatric patients from Finland, the authors observed that lower plasma levels of n-3 fatty acids were linked to higher plasma levels of neurosteroids. Specifically, reduced concentrations of DHA and EPA were correlated with increased levels of NASs in healthy control subjects [158]. DHA supplementation reduced stress-related increases in aggression and hostility among Japanese students [159]. The research conducted by Oravcova et al. [160] on patients aged 11–18 confirmed the hypothesis that long-term (12 weeks) supplementation of n-3 fatty acids from fish oil emulsion reduces morning cortisol levels in saliva. These findings are in line with the current understanding of the connection between HPA axis activity and fatty acid metabolism in adults with recurrent depressive disorder [155]. Adults with lower levels of n-3 fatty acids demonstrated HPA axis dysregulation, suggesting that supplementation with these fatty acids could potentially improve both physical and mental health [161]. Notably, dietary supplementation with LC-PUFAs from marine oils has been shown to lower corticosterone levels in rats and to reduce cortisol secretion in both healthy individuals and adults experiencing depression [151,162,163]. Results from a randomized, placebo-controlled trial in midlife adults demonstrated that EPA and DHA supplementation significantly reduced cortisol levels during stress in a dose–response manner. Patients receiving the higher doses of n-3 fatty acids had the lowest overall cortisol levels, while those in the placebo group exhibited the highest levels [164]. Additionally, supplementation with n-3 fatty acids (2400 mg of total omega-3 fatty acids; 1000 mg of EPA and 750 mg of DHA; EPA:DHA ratio of 1.33:1) over a 12-week period was shown to significantly reduce clinical symptoms of depression in older children and adolescents [165]. In adolescent patients, the positive treatment effects from n-3 fatty acid supplementation were also associated with a decrease in various oxidative stress markers, such as 8-isoprostane, advanced oxidation protein products, and nitrotyrosine blood levels, as well as increased Trolox equivalent antioxidant capacity and superoxide dismutase activity [166].

Chronic stress or inflammation can result in the overactivation of the HPA axis. Previous studies have demonstrated increased levels of inflammatory markers, including IL-6 and TNF-α, in numerous chronic diseases such as cardiovascular diseases, obesity, and depression, contributing to their development and progression [167,168,169]. Additionally, HPA axis hyperactivity has been observed in obesity, and increased morning cortisol levels have been reported in individuals with depression [170,171]. Given their anti-inflammatory and immunomodulatory effects, n-3 PUFAs may help mitigate these detrimental processes and thereby potentially alleviate conditions characterized by HPA axis dysregulation [172,173]. n-3 fatty acids can lower HPA axis hyperactivity by decreasing proinflammatory cytokines levels, such as IL-6 and TNF-α, which influence the stress response. By downregulating the proinflammatory cytokine pathways and modulating the stress response, n-3 fatty acids reduce the sensitivity of the HPA axis. This means that the axis is less likely to become overactive in response to minor stressors, potentially lowering the risk of stress-related disorders. In a randomized, double blind, placebo-controlled trial conduced on healthy man, n-3 supplementation in combination with phosphatidylserine significantly improved the functioning of the HPA axis by lowering chronic and acute stress [174]. The authors concluded that individuals experiencing high levels of chronic stress and/or having a dysfunctional HPA axis response could benefit from n-3 phosphatidylserine supplementation. The impact of n-3 fatty acids on regulating the activity of the HPA axis may be related to their anti-inflammatory properties, coupled with the increase in the HPA axis sensitivity to negative feedback [175,176].

### 3.5. Modulation of the Gut Microbiota by Omega-3 Fatty Acids

The beneficial physiological effects of LC-PUFAs encompass their influence on the composition and function of the gut microbiota, as demonstrated in various studies linking fatty acid intake to microbial diversity and health outcomes. n-3 fatty acids can influence the modulation and abundance of gut bacteria types. At the same time, gut microbiota can affect the absorption and metabolism of these fatty acids. Fish oil, for instance, reduces the growth of *Enterobacteria*, while increasing that of *Bifidobacteria*. EPA and DHA are partially metabolized by anaerobic bacteria, such as *Bifidobacteria* and *Lactobacilli*, in the distal gut [177]. In a study conducted on gnotobiotic piglets fed with PUFA from seal oil, a significant increase in the number of *Lactobacillus paracasei* adhering to the jejunal mucosa was shown [178]. Similarly, n-3 fatty acids administered to male transgenic fat-1 mice significantly increased the number and percentage of *Bifidobacterium*, *Akkermansia muciniphila*, *Lactobacillus*, *Clostridium clusters IV* and *XIVa*, and *Enterococcus faecium* in the intestines [179]. The modulatory functions of n-3 fatty acids are also attributed to increased levels of SCFAs. It has been shown that dietary supplementation with DHA and EPA in mice infected with *Salmonella* increased SCFA fecal content, enhancing resistance against the pathogen [180]. Consuming 3 g per day of DHA and EPA from sardines significantly altered the gut microbiota composition in patients with untreated type 2 diabetes. Specifically, the proportions of *Bacteroides*/*Prevotella* increased, while the *Firmicutes/Bacteroidetes* ratio decreased [181]. In a study of birds, a diet rich in omega-3 PUFA significantly increased the presence of *Firmicutes* (e.g., *Faecalibacterium*, *Clostridium*, and *Ruminococcus*, all of which are butyrate producers), in the gut microbiota [182]. Additionally, serum levels of omega-3 fatty acids, particularly DHA, were positively correlated with gut microbiome diversity and the abundance of specific bacterial taxa, such as *Lachnospiraceae*, in a cohort of 876 elderly women, suggesting a potential role for LC-PUFA intake in modulating microbiome composition [183]. A 6-week intervention study further demonstrated that omega-3 supplementation alters gut microbiome composition, increasing the abundance of butyrate-associated *Coprococcus* spp. and beneficial fermentation products, suggesting a potential prebiotic-like role for omega-3 fatty acids [184].

On the other hand, gut microbiota can both indirectly and directly modulate the absorption, bioavailability, and biotransformation of n-3 fatty acids [185,186,187]. Certain bacterial species, such as *Bacillus proteus* or *Lactobacillus plantarum*, convert ALA and linoleic acid (LA) into conjugated linoleic acid (CLA) and conjugated alpha-linolenic acid (CALA), which are then hydrogenated to stearic acid, changing the PUFA content in the brain and heart [188]. A study of mice demonstrated that high tissue levels of n-3 fatty acids were associated with variations in the amounts of *Bifidobacterium* and *Lactobacillus* [189]. Conversely, mice fed a diet low in n-3 fatty acids for two generations showed a significant decrease in lactic acid bacteria and an increase in *Bifidobacteria* in the oral cavity compared to those fed a diet adequate in n-3 fatty acids [190]. While studies suggest that *Bifidobacterium* can significantly modulate fatty acid metabolism and its absorption by the intestinal epithelium, specific mechanisms underlying this relationship remain unexplained [191,192,193].

n-3 Fatty acids can modulate gut microbiota by either inhibiting the production of proinflammatory mediators or promoting the production of anti-inflammatory mediators. For instance, DHA reduces its activation by lowering IkappaB kinase (IκB) phosphorylation in response to LPS in cultured macrophages and dendritic cells, thereby decreasing NFκB activation [194,195]. Similarly, EPA reduced NFκB activation induced by LPS in human monocytes due to reduced IκB phosphorylation [196]. Furthermore, peroxisome proliferator-activated receptor gamma (PPAR-γ), which functions by inhibiting NFκB translocation to the nucleus, is regulated by the binding of EPA and DHA [197]. This binding interaction significantly influences inflammatory processes [198]. Supplementation with these acids significantly influences changes in the gut microbiota by altering the content of immune cell membranes and influencing proinflammatory signaling pathways. n-3 Fatty acids increase the number of regulatory T lymphocytes (Tregs), thereby reducing inflammatory reactions [199]. n-3 Fatty acids are mainly absorbed in the intestine, where their metabolites can be directly utilized by certain microorganisms. Their protective action on the intestinal mucosa includes increasing its thickness and improving barrier functions [200]. Summarized data presenting the mechanistic effects of LC-PUFAs on gut microbiota, immune function, and intestinal barrier integrity are presented in Table 2.

## 4. Conclusions

**Figure 4:**
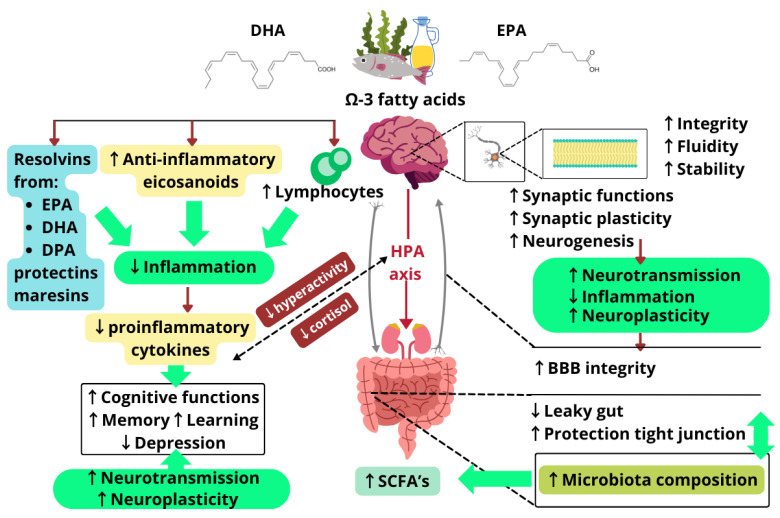
Summary of n-3 PUFAs’ mechanisms of action: a schematic representation of the possible mechanisms through which n-3 PUFAs influence the gut–brain axis. DHA—docosahexaenoic acid; EPA—eicosapentaenoic acid; DPA—n-3 docosapentaenoic acid; HPA—hypothalamic-pituitary-adrenal; BBB—blood–brain barrier; SCFAs—short-chain fatty acids. ↑ indicates an increase, ↓ indicates a decrease. Designed using elements by ©Canva, sparklestroke, Pixeden, iconsy, OpenClipart-Vectors via Canva.com (access date: 18 November 2024).

n-3 Fatty acids, particularly EPA and DHA, play a pivotal role in the intricate communication network of the GBA. Their incorporation into cell membranes enhances membrane fluidity, which is essential for optimal neurotransmitter function and efficient signal transduction. By modulating inflammatory responses—reducing proinflammatory cytokines and promoting anti-inflammatory mediators—n-3 fatty acids help preserve the integrity of critical barriers, like the intestinal barrier and the BBB. This not only supports gut health but also protects the CNS from potential neurotoxins and inflammatory agents. In the CNS, EPA and DHA contribute to neurogenesis and synaptic plasticity, thereby enhancing cognitive functions such as learning and memory. They also regulate the HPA axis by mitigating excessive cortisol production, which is often associated with stress responses and mental health disorders, like depression and anxiety. Furthermore, n-3 fatty acids positively influence gut microbiota composition, promoting growth of beneficial bacterial populations that contribute to gut health and systemic immunity. The effects of n-3 fatty acids on the GBA are summarized in Figure 4.

Given the multifaceted benefits of n-3 fatty acids on the GBA, it is recommended to incorporate adequate amounts of EPA and DHA into the diet by consuming marine foods, like fatty fish, or through supplementation. Additionally, based on the significant benefits of n-3 fatty acids on the GBA and overall health, the following nutritional recommendations are proposed:

1. Incorporate fish rich in n-3 fatty acids, such as salmon, mackerel, sardines, trout, and herring, into the diet at least twice a week to boost EPA and DHA intake according to recommendation to achieve essentiality and cardiovascular benefits [201].
2. Consider n-3 fatty acid supplements based on fish oil or algae, especially for individuals with limited access to n-3-rich foods or with dietary restrictions [202].
3. Consume fiber-rich foods, like whole grains, fruits, and vegetables, along with fermented foods like yogurt, kefir, and sauerkraut, to support beneficial gut microbiota that synergizes with n-3 fatty acids [203].
4. Use cooking methods that preserve n-3 content, such as baking, steaming, or grilling, and avoid high-temperature frying, which can oxidize these delicate fats [204].
5. To ensure adequate intake of omega-3 fatty acids, individuals are encouraged to follow national or international dietary guidelines, such as those provided by the World Health Organization, the Institute of Medicine, or the European Food Safety Authority, which offer evidence-based recommendations for maintaining optimal health.

## 5. Limitations and Current Knowledge Gaps

Despite the existing research on the influence of LC-PUFAs on the GBA, critical gaps in our understanding of the precise mechanisms underlying this interaction persist. For instance, the exact processes by which n-3 PUFAs stabilize physiological barriers—such as the BBB and the intestinal barrier—and how they modulate the activity of both immune and neuronal cells, remain incompletely elucidated. Furthermore, the complex relationships between gut microbiota composition, n-3 fatty acid metabolism, and the regulation of inflammatory states require more investigations to fully clarify their interconnected roles. Despite substantial evidence demonstrating the beneficial effects of LC-PUFAs on the GBA, the major limitation is the difficulty of extrapolating doses and outcomes from in vitro and animal models to human populations. The concentrations of fatty acids used in cell culture studies and the dietary interventions used in animal experiments often exceed physiologically achievable levels in human.

## Figures and Tables

**Table 2:** Summarized effects of LC-PUFAs on the gut microbiota, immune function, and intestinal barrier integrity.

Models	Type of Study	Source and Dose of n-3 PUFA	Exposure	Effect	Reference
In vitro model studies					
RAW 264.7 murine macrophage-like cell line	in vitro	100 μM DHA	24 h	- DHA reduced NFκB-DNA binding activity - Reduced inflammation	[194]
Murine bone marrow-derived DC	in vitro	100 μM DHA	24 h	- Reduction NFκB translocation mediated by inhibition of IκB degradation - Reduced inflammation	[195]
RAW 264.7 murine MØ cell line	in vitro	12 mg%, ω-3 FA emulsion	4 h	- Reduction in endotoxin-induced NFκB activation through decreased IκB phosphorylation - Reduced inflammation	[198]
Human Jurkat T cell lines E6-1	in vitro	50 μm EPA	48 h	- Promotion of regulatory T lymphocyte (Treg) induction and prevention of excessive development of T helper 17 (Th17) cells increase the number of regulatory T lymphocytes (Tregs) - Reduced inflammation	[199]
In vivo model and human subject studies					
Male BALB/c mice with chronic stress	in vivo	Squid egg and sea cucumber (9% EPA and 38.9% DHA or 36% EPA and 5% DHA or 79% EPA and 10% DHA)	21 days	- Increase *Lactobacillus*, *Prevotella* spp., *Bacteroides fragilis*, and *Roseburia* spp. - Decrease *Enterobacteriaceae* and *Enterococcus* spp. - Protection against intestinal dysfunction - Attenuation of proinflammatory processes - Amelioration of LPS increase	[177]
Poultry	in vivo	0.2% and 0.6% of total n-3 PUFA in the diet (marine algal biomass or flaxseed oil)	8 weeks	- Increased population of *Firmicutes* (e.g., *Faecalibacterium*, *Clostridium* and *Ruminococcus*	[182]
C57BL/6J female mice were and their male offspring	in vivo	∼1 g EPADHA/100 g of the diet	12 weeks	- Increased fecal *Bifidobacterium* and *Lactobacillus* abundance in offspring	[189]
Male Sprague Dawley rats with intestinal damage	in vivo	300 μg/kg per day (EPA 180 μg + DHA 120 μg)	Once per day 48 h before and 72 h after MTX injection	- Increased mass of the colon and ileum - Greater mass of the ileal mucosa—increased villus height and crypt depth in the ileum	[200]
Drug-naïve patients with type 2 diabetes	human subjects	3.0 ± 0.2 g EPA + DHA/d (sardines)	5 days a week for 6 months	- Increased ratio of *Bacteroides*/*Prevotella* - Decreased ratio of *Firmicutes*/*Bacteroidetes*	[181]

## Data Availability

Not applicable.
